# The absence of reactive oxygen species production protects mice against bleomycin-induced pulmonary fibrosis

**DOI:** 10.1186/1465-9921-6-11

**Published:** 2005-01-21

**Authors:** Boris Manoury, Soazig Nenan, Olivier Leclerc, Isabelle Guenon, Elisabeth Boichot, Jean-Michel Planquois, Claude P Bertrand, Vincent Lagente

**Affiliations:** 1 Laboratoire de Pharmacodynamie et de Pharmacologie Moléculaire, INSERM U620, Université de Rennes 1, Rennes, France; 2 Pfizer Global R&D, Fresnes Laboratories, Fresnes, France

## Abstract

**Background:**

Reactive oxygen species and tissue remodeling regulators, such as metalloproteinases (MMPs) and their inhibitors (TIMPs), are thought to be involved in the development of pulmonary fibrosis. We investigated these factors in the fibrotic response to bleomycin of p47^phox ^-/- (KO) mice, deficient for ROS production through the NADPH-oxidase pathway.

**Methods:**

Mice are administered by intranasal instillation of 0.1 mg bleomycin. Either 24 h or 14 days after, mice were anesthetized and underwent either bronchoalveolar lavage (BAL) or lung removal.

**Results:**

BAL cells from bleomycin treated WT mice showed enhanced ROS production after PMA stimulation, whereas no change was observed with BAL cells from p47^phox ^-/- mice. At day 1, the bleomycin-induced acute inflammatory response (increased neutrophil count and MMP-9 activity in the BAL fluid) was strikingly greater in KO than wild-type (WT) mice, while IL-6 levels increased significantly more in the latter. Hydroxyproline assays in the lung tissue 14 days after bleomycin administration revealed the absence of collagen deposition in the lungs of the KO mice, which had significantly lower hydroxyproline levels than the WT mice. The MMP-9/TIMP-1 ratio did not change at day 1 after bleomycin administration in WT mice, but increased significantly in the KO mice. By day 14, the ratio fell significantly from baseline in both strains, but more in the WT than KO strains.

**Conclusions:**

These results suggest that NADPH-oxidase-derived ROS are essential to the development of pulmonary fibrosis. The absence of collagen deposition in KO mice seems to be associated with an elevated MMP-9/TIMP-1 ratio in the lungs. This finding highlights the importance of metalloproteinases and protease/anti-protease imbalances in pulmonary fibrosis.

## Background

Pulmonary fibrosis is a severe chronic disease with various causes and poor prognosis. Its main histological features include lesions of the alveolar septa, fibroblast and myofibroblast proliferation in lung parenchyma, abnormal reepithelialization, and excessive extracellular matrix macromolecule deposition [1-3]. Lung fibrosis is associated with chronic inflammation and is characterized by the recruitment of macrophages, neutrophils, and lymphocytes in the airways [4]. During lung inflammation, activated phagocytes release large amounts of reactive oxygen species (ROS), which may be involved in tissue injury and in impeding tissue repair, both of which lead to pulmonary fibrosis [4-6]. Recent studies show that antioxidant compounds such as N-acetylcysteine and bilirubin protect rats against the tissue damage and pulmonary fibrosis induced by bleomycin, an antineoplastic antibiotic commonly used in such experimental models [7,8]. Because these compounds can attenuate the oxidant burden in tissue, they may prevent the lung damage caused by ROS and subsequent fibrosis.

Metalloproteinases (MMPs) and their specific inhibitors, the tissue inhibitors of MMPs (TIMPs), are the hallmark of this fibrogenic microenvironment. MMPs are key enzymes that regulate tissue remodeling through turnover of the extracellular matrix in both normal and pathological conditions (for review see [9]). They play a crucial role in the fibrogenic process, as demonstrated recently through the marked reduction of bleomycin-induced pulmonary fibrosis in mice by batimastat, a selective MMP inhibitor [10]. Gelatinase A (MMP-2) and gelatinase B (MMP-9) are two MMPs that appear to be involved in pulmonary fibrosis, but their specific roles in the process remain unclear [9]. While MMP-9 is released primarily by inflammatory cells, MMP-2 is synthesized by structural cells including fibroblasts and endothelial and epithelial cells. Both may be associated with chronic impairment of tissue remodeling and abnormal collagen deposition [9].

Strong evidence indicates that various MMP/TIMP imbalances are crucial elements in the fibrogenic process. Several authors suggest that a "nondegrading microenvironment" induces fibrogenicity, that is, more specifically, that various events cause TIMP-1 levels to rise in lung tissue, which in turn lowers MMP/TIMP ratios [2,11,12]. Bleomycin-induced pulmonary fibrosis, for example, causes the expression of significant levels of TIMP-1 [13,14].

Further study is needed to illuminate the pathway that leads from lung injury, associated with ROS and acute inflammation, to initiation of the fibrogenic process, which involves remodeling mediators such as MMPs and TIMPs. The aim of the present study was to investigate the involvement of the ROS released by inflammatory cells during the development of pulmonary fibrosis and to consider the consequence on MMP/TIMP balances. We therefore examined the fibrogenic response to bleomycin administration in mice deficient for the p47^phox ^subunit of NADPH-oxidase [15] and analyzed the variations in the MMP/TIMP balance during this process.

## Materials and methods

### Materials

This study used the following materials, from the manufacturers mentioned: bleomycin sulfate from Bellon Laboratories (Montrouge, France); gelatin, Triton X-100, Coomassie Brilliant Blue, EDTA, Tween 20 solution, hydroxyproline, and trypan blue from Sigma (St Louis, MO, USA); May-Grünwald and Giemsa stains from RAL (Paris, France); sodium pentobarbital from Sanofi Santé Animale (Libourne, France); etomidate (Hypnomidate^®^, 2 mg/mL) from Janssen-Cilag (Issy-les-Moulineaux, France); acrylamide, sodium dodecyl sulfate (SDS), Tris, and BSA from Eurobio (Les Ulis, France); ELISA kits for IL-6, TIMP-1, and pro-MMP-9 detection from R&D Systems (Minneapolis, MN, USA); formaldehyde from Merck (Darmstadt, Germany); isopentane from Prolabo (Fontenay-sous-Bois, France); a low-range weight marker for SDS-PAGE from Biorad (Munich, Germany); and an ABEL^® ^chemiluminescence kit for measurement of *in vitro *ROS release from Knight Scientific Limited (Plymouth, UK).

### Bleomycin administration

Ten week-old p47^phox ^+/+ "wild-type" (WT) and p47^phox ^-/- "knockout" (KO) mice (origin: LHD/NIAID/NIH, Bethesda, MD, USA) with C57BL/6J backgrounds [15] were housed under controlled and ethical conditions that complied with the Interdisciplinary Principles and Guidelines for the Use of Animals in Research, Marketing and Education, New York Academy of Sciences' Ad Hoc Committee on Animal Research.

Pulmonary fibrosis was induced by intranasal (i.n.) instillation (under etomidate anesthesia, 15 mg i.p.) of 0.1 mg bleomycin sulfate in saline solution (50 μL/mouse). Control mice received saline vehicle only. Either 24 h or 14 days after i.n. administration, mice were quickly anesthetized by an i.p. injection of sodium pentobarbital (60 mg/kg) and underwent either bronchoalveolar lavage (BAL) or lung removal. These samples were stored at -80°C until either hydroxyproline measurement or homogenization for zymography.

### Bronchoalveolar lavage and preparation of tissue homogenates

Mice were anesthetized with an i.p. administration (20 mL/kg) of sodium pentobarbital 0.6%. The BAL protocol called for washing the airways 10 times with 0.5 mL of 0.9% NaCl solution at 37°C with a 1 mL syringe. The BAL fluid was centrifuged (600 g for 10 min, 4°C), and the supernatant of the first two fractions (1 mL) divided into aliquots and frozen at -80°C until analysis. The cell pellets were then pooled with the last fractions. Total cells were counted with a Coulter Z2^® ^(particle counter and size distribution analyzer, Beckman Coulter). Red blood cells were eliminated by adding 3 mL of distilled water for 30 seconds and then 1 mL of KCl 0.6 M onto the pellets. After centrifugation (600 g for 10 min, 4°C), supernatant was eliminated and the cells were suspended in 1 mL of PBS. They were then cytospun at 700 rpm for 10 minutes (Cytospin 3 ^®^, Thermo Shandon, Ltd, Astmoor, United Kingdom) and stained with the May-Grünwald Giemsa method. Differential cell counts of 200 cells used standard morphological criteria.

After in vitro stimulation with phorbol 12-myristate 13-acetate (PMA), 0.8 μM, ROS production was assayed with a chemiluminescence technique that used the ABEL^® ^detection kit.

At day 14, following BAL processing, lungs were removed and homogenized with an adapted grinder (Fast-Prep FP 120 cell disrupter, QBiogene Inc., Illkirch, France). Lung tissue homogenates were then stored at -80°C until analysis.

### Zymographic analysis of MMPs

Since MMPs can degrade gelatin, zymographic techniques were used to detect MMPs in both BAL (day 1 and day 14) and lung homogenates (day 14). In nonreducing conditions and in the presence of SDS, as previously described [10], aliquots of BAL fluid or lung homogenate underwent electrophoresis onto a 6% acrylamide stacking gel/10% acrylamide separating gel containing 1 mg/mL gelatin. After electrophoresis, gels were washed twice with 2.5% Triton X-100, rinsed with water, and incubated overnight at 37°C in 50 mM Tris, 5 mM CaCl_2_, 2 μM ZnCl2, pH = 8. The gels were stained with Coomassie Brilliant Blue in a solution of 25% ethanol-10% acetic acid in water and rinsed in an identical solution. Gelatinase activity appeared as clear bands against blue background. We used recombinant protein molecular weight markers (20 kDa-112 kDa) to estimate the molecular weights of the gelatinolytic bands. Relative enzyme amounts were quantified by measuring the intensity of the bands with a densitometric analyzer (Bio-Profile, Vilber-Lourmat, Marne la Vallée, France). Results were expressed as a percentage of the band of migration of one control BAL sample loaded onto each gel. This sample was used as an internal standard to allow further comparisons between gels.

### Determination of IL-6, TIMP-1 and pro-MMP9 levels in BAL

The amounts of total IL-6, TIMP-1, and pro-MMP-9 were determined with ELISA methods, performed according to the manufacturer's recommendations. Assay sensitivity was 31 pg/mL for TIMP-1, 15 pg/mL for IL-6, and 8 pg/mL for pro-MMP-9.

### Hydroxyproline measurement

Lung tissue was lyophilized, weighed, ground to a fine powder with a mortar and pestle, homogenized in PBS pH = 7.4, and divided into aliquots. After hydrolysis for 45 min in NaOH 2 N, the hydroxyproline content was assayed in duplicate aliquots, as previously described [16].

### Expression of the results and statistical analysis

Results were expressed as means ± SEM. The differences between the groups for treatment and strain effects were analyzed with a nonparametric Mann-Whitney U test. Correlations between the BAL analysis data and the MMP levels and activity were assessed with the nonparametric Spearman correlation test. For each analysis, P values less than 0.05 were considered to be statistically significant.

## Results

### Reactive oxygen species production and cell recruitment in BAL fluids

**Figure 1:**
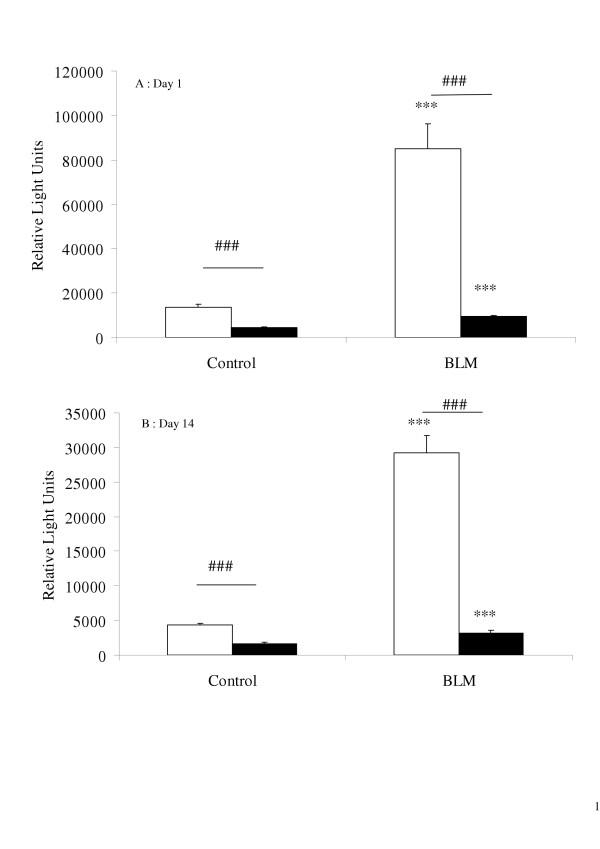
Production of reactive oxygen species (ROS) by bronchoalveolar lavage (BAL) cells, 1 day (A) and 14 days (B) after intranasal administration of bleomycin (0.1 mg, BLM) or saline (Control) to mice with the p47^phox ^subunit of NADPH-oxidase deleted (p47^phox ^-/- knockout mice (KO): solid bars), compared with "Wild Type" p47^phox ^+/+ mice (WT: blank bars). Cells were stimulated *in vitro *with PMA (0.8 μM) and ROS production was evaluated by chemiluminescence. Data were collected at the time of maximum light emission. Results are expressed as relative light units (mean ± SEM). ***: p < 0.001 comparison with control mice exposed to saline solution alone. ###: p < 0.001 for KO mice compared with WT mice. n = 5–9.

At both day 1 and day 14, bleomycin treatment elicited enhanced ROS production by BAL cells from WT mice stimulated *in vitro *with PMA (Figure 1). In contrast, neither the cells removed from control mice (WT, not treated with bleomycin) nor those from KO mice (regardless of bleomycin treatment) could produce ROS *in vitro *after PMA stimulation. No chemiluminescence was detected when BAL cells were not stimulated by PMA *in vitro*.

At day 1, ROS release by BAL cells from bleomycin-treated WT mice was accompanied by a significant neutrophil influx, but the total BAL cell count did not rise (Table 1). This bleomycin-induced neutrophil influx was quite noticeable in BAL from KO mice, significantly greater than in WT mice.

**Table 1:** Total and differential cell counts of BAL fluid from p47^phox ^+/+ WT and p47^phox ^-/- KO mice, at day 1 and day 14 after intranasal administration of bleomycin (BLM, 0.1 mg/mouse) or saline vehicle (Control, NaCl 0.9%). Results are presented as the mean (.10^3^cells) ± SEM. n: number of mice. WT: wild-type; KO: knockout; a: P < 0.05, b: P < 0.01, c: P < 0.001 compared with control mice exposed to saline solution only. *: P < 0.05, **: P < 0.01, ***: P < 0.001 for KO mice compared with WT mice.

Treatment	Strain	N	Total Cells	Macrophages	Neutrophils	Eosinophils	Lymphocytes
Control	[WT]	10	540 ± 99	536 ± 97	4 ± 2	0	0
	[KO]	10	535 ± 105	393 ± 69	129 ± 37 ***	1 ± 1	11 ± 5 **

BLM, day 1	[WT]	8	490 ± 77	317 ± 50	164 ± 28 ^c^	0	0
	[KO]	9	2208 ± 733 ^b, e^	281 ± 102	1922 ± 634 ^c, ^**	0	2 ± 2

BLM, day 14	[WT]	8	1108 ± 80 ^b^	991 ± 77 ^a^	76 ± 15 ^c^	20 ± 6 ^b^	24 ± 13 ^b^
	[KO]	9	793 ± 118	634 ± 97 ^a, ^*	62 ± 12	18 ± 16	80 ± 33 ^a^

Fourteen days after bleomycin administration, the total cell count in the WT mouse BAL increased significantly. Specifically, the alveolar macrophage count rose markedly, as did the neutrophil, eosinophil, and lymphocyte counts, although to a lesser extent. The total cell count in the BAL fluid of the KO mice did not increase significantly, but the number of macrophages and lymphocytes did. The WT mice had significantly more alveolar macrophages than the KO mice (Table 1).

### Lung hydroxyproline measurement

Lung hydroxyproline concentration, which reflects collagen deposition in lungs, was measured 14 days after bleomycin administration to quantify pulmonary fibrosis (Table 2). Hydroxyproline levels did not increase in the KO mice. Moreover, although the hydroxyproline level was similar in both strains of mice at baseline, it was significantly higher in WT than in KO mice at day 14.

**Table 2:** Hydroxyproline content (mg/g of dry tissue) in lung homogenate from p47^phox ^+/+ WT and p47^phox ^-/- KO mice, at day 14 after intranasal administration of bleomycin (BLM, 0.1 mg/mouse) or saline vehicle (Control, NaCl 0.9%). Results are presented as mean ± SEM. WT: wild-type; KO: knockout; ** : P < 0.01 for WT mice compared with KO mice. n = 3–6.

Strain	Control	BLM
p47^phox ^+/+ [WT]	1.20 ± 0.26	1.74 ± 0.10**
p47^phox ^-/- [KO]	1.29 ± 0.24	1.04 ± 0.10

### IL-6 levels in BAL fluids

**Figure 2:**
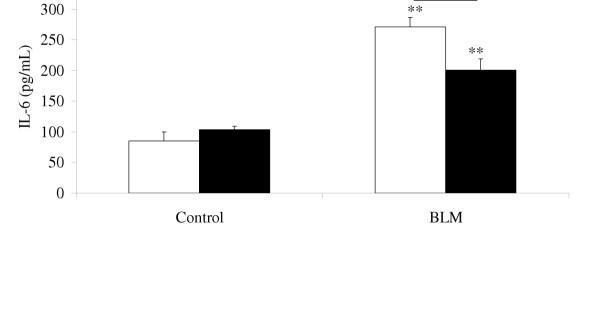
Level of IL-6 (pg/mL) in BAL fluids, 1 day after intranasal administration of bleomycin (BLM, 0.1 mg/mouse) or saline vehicle (Control), to p47^phox ^+/+ WT mice (blank bars) and p47^phox ^-/- KO mice (solid bars). Results are presented as the mean ± SEM. ** : p < 0.01 compared with control mice exposed to saline solution alone. #: p < 0.05 for p47^phox ^-/- KO mice compared with p47^phox ^+/+ WT mice. n = 4–9.

IL-6 levels in the BAL fluids of WT and KO mice rose one day after bleomycin administration (figure 2) and were significantly higher in WT than KO mice. Fourteen days after bleomycin administration, IL-6 levels were not significantly different from baseline.

### MMP activity in BAL fluids and lung homogenates

**Figure 3:**
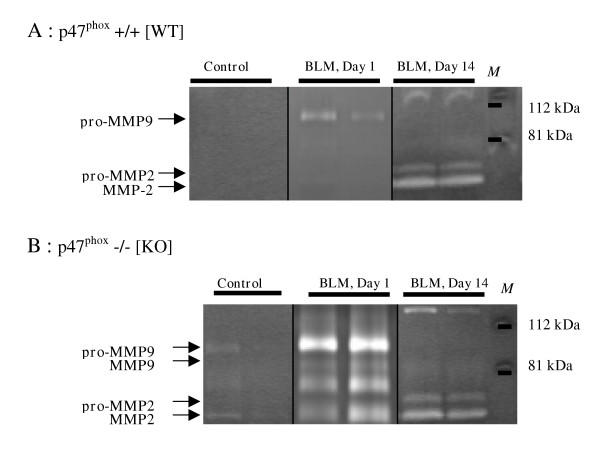
Representative gelatin zymogram of BAL supernatant fluids, 1 and 14 days after intranasal administration of bleomycin (BLM, 0.1 mg/mouse) or saline vehicle (Control) to p47^phox ^+/+ WT mice (A) and p47^phox ^-/- KO mice (B). The following gelatinolytic bands were identified as MMP activity: pro-MMP-9 (105 kDa), MMP-9 (86 kDa), pro-MMP-2 (70 kDa), and MMP-2 (64 kDa). M: molecular weight marker.

**Figure 4:**
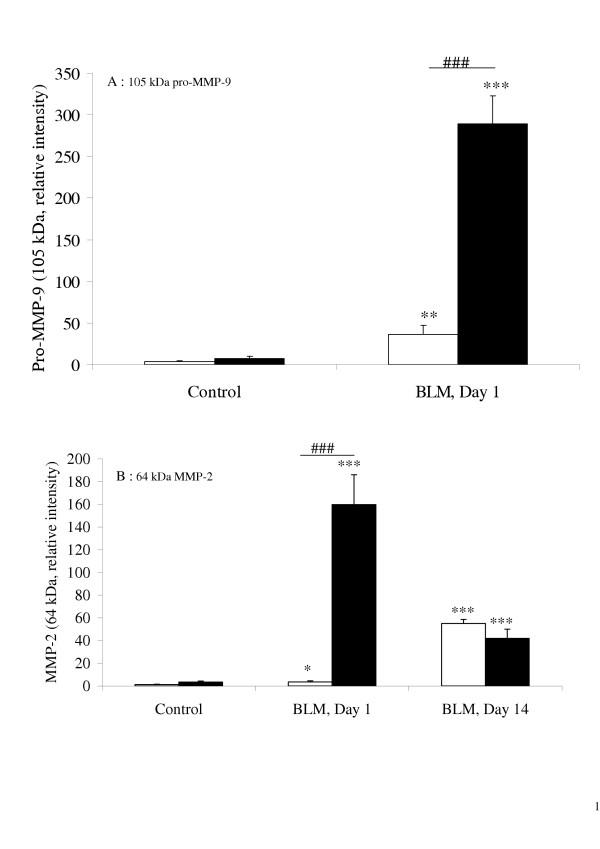
Quantification by densitometry of 105-kDa pro-MMP-9 (A), and 64-kDa MMP-2 (B) gelatinase activity on zymograms of BAL fluid, performed 1 day or 14 days after intranasal administration of bleomycin (BLM, 0.1 mg/mouse) or saline vehicle (Control), to p47^phox ^+/+ WT mice (blank bars) and p47^phox ^-/- KO mice (solid bars). Results are represented as the mean of relative intensity ± SEM. * : p < 0.05, ** : p < 0.01, *** : p < 0.001 compared with control mice exposed to saline solution alone. ###: p < 0.001 for p47^phox ^-/- KO mice compared with p47^phox ^+/+ WT mice. n = 8–10.

Zymography identified the following gelatinolytic bands as MMP activity: pro-MMP-9 (105 kDa), MMP-9 (86 kDa), pro-MMP-2 (70 kDa), and MMP-2 (64 kDa). At day one, pro-MMP-9 activity was significantly higher in the BAL of bleomycin-treated KO mice than in that of their bleomycin-treated WT counterparts, which in turn was significantly higher than in the control mouse BAL (figure 3 and figure 4A). At day 14, no pro-MMP-9 activity was observed in any of the mice. The active form of MMP-9 (86 kDa) was detected only in KO mouse BAL fluid at day 1 (figure 3). Pro-MMP-9 was significantly correlated with the neutrophil count in the BAL fluids of both WT (P = 0.001) and KO (P = 7 × 10^-6^) mice.

At one day and 14 days after bleomycin administration, MMP-2 activity was observed in both its latent (70 kDa) and active (64 kDa) forms (figure 3). Although densitometry analysis could detect only the 64-kD form (figure 4B) at day one, bleomycin elicited a significant increase in the 64-kDa MMP-2 activity in both strains, compared with the control; and this activity was substantially stronger in the BAL of KO than WT mice. At day 14, MMP-2 activity remained higher in both strains of bleomycin-treated mice (compared with controls) and did not differ significantly between them (figure 4B).

**Figure 5:**
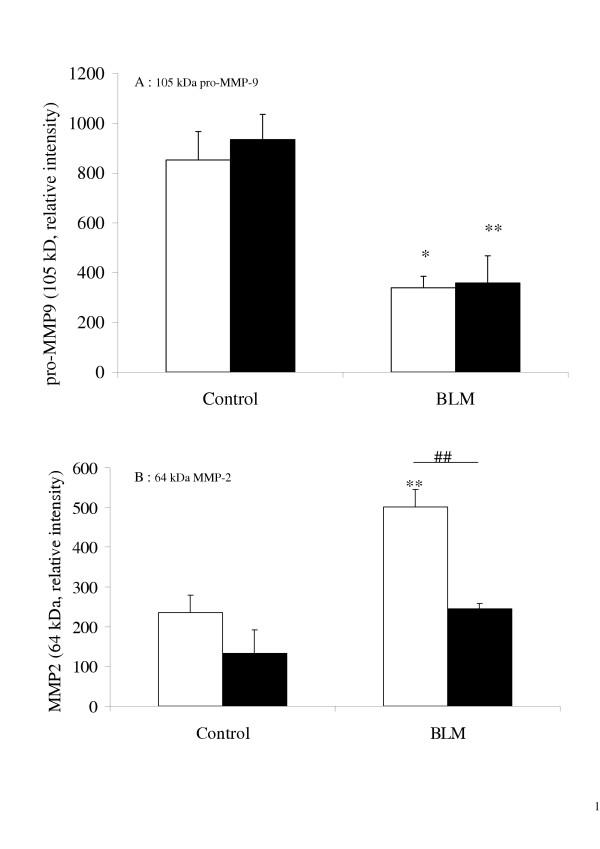
Quantification by densitometry of Pro-MMP-9 (105 kDa, A) and MMP-2 (64 kDa, B) gelatinase activity on zymograms of lung homogenates, performed 14 days after intranasal administration of bleomycin (BLM, 0.1 mg/mouse) or saline vehicle (Control) to p47^phox ^-/- KO mice (solid bars) and p47^phox ^+/+ WT mice (blank bars). Results are represented as the mean of relative intensity ± SEM. * : p < 0.05, ** : p < 0.01, compared with control mice exposed to saline solution alone. ##: p < 0.01 for p47^phox ^-/- [KO] mice compared with p47^phox ^+/+ WT mice. n = 3–5.

MMP activity was also evaluated at day 14 in lung homogenates (figure 5). Homogenate from both WT and KO mice showed similar levels of pro-MMP-9 activity levels, unexpected lower than in the homogenate from the control mice. In contrast, MMP-2 activity increased in the lungs of WT mice only (figure 5).

### Pro-MMP-9/TIMP-1 ratio in BAL fluids

We also evaluated pro-MMP-9 as well as TIMP-1 with ELISA (table 3). The results for pro-MMP-9 confirm those obtained with zymography. In WT mice, TIMP-1 in BAL fluid was markedly higher at day one and day 14 after bleomycin administration than at baseline. In contrast, the TIMP-1 level in the BAL fluid of KO mice was not significantly modified by bleomycin administration at either day 1 or day 14 (table 3).

**Table 3:** Levels of pro-MMP-9 and TIMP-1, and pro-MMP-9 / TIMP-1 ratio in BAL supernatant fluids, recovered from p47^phox ^+/+ [WT] and p47^phox ^-/- [KO] mice, 1 day or 14 days after intranasal administration of bleomycin (BLM, 0.1 mg/mouse) or saline vehicle (Control, NaCl 0.9%). For Pro-MMP-9 and TIMP-1, results are represented as the mean (pg/ml) ± SEM. N : number of mice. a : P < 0.05, b : P < 0.01, in comparison to control mice exposed to saline solution only. * : P < 0.05, ** : P < 0.01, for [KO] mice in comparison to [WT] mice.

Treatment	Strain	N	Pro-MMP-9	TIMP-1	Ratio Pro-MMP-9 / TIMP-1
Control	[WT]	5	46.3 ± 14.9	38.7 ± 13.5	2.7 ± 1.3
	[KO]	5	1314.7 ± 798.2 **	325.2 ± 173.6	10.4 ± 4.7

BLM, day 1	[WT]	7	2645.9 ± 1 011.6 ^b^	781.8 ± 158.9 ^b^	3.8 ± 1.3
	[KO]	8	23646.8 ± 2 359.7 ^b, ^**	1 213.8 ± 396.4	60.8 ± 23.7 ^a, ^**

BLM, day 14	[WT]	7	37.7 ± 9.1	2693.3 ± 378.3 ^b^	0.02 ± 0.00 b
	[KO]	6	137.9 ± 15.5 ^b^	567.9 ± 163.5**	0.2 ± 0.1 ^b, ^**

Table 3 presents the calculation of the pro-MMP-9/TIMP-1 ratio. At day one after bleomycin administration, this ratio remained stable in the BAL of WT mice, both treated and untreated, but rose significantly in that of the KO mice (table 3). At day 14, the ratio was significantly lower in both strains of treated mice than in the control mice, and levels in the WT mice were significantly lower than those in the KO mice.

## Discussion

This study shows that mice deficient in the p47^phox ^subunit of the NADPH oxidase complex do not develop pulmonary fibrosis after intranasal administration of bleomycin. It also suggests that an imbalance of the molar MMP-9/TIMP-1 ratio may influence the fibrogenic process in this model.

Several studies previously reported that antioxidant treatment attenuates the bleomycin-induced oxidative burden and subsequent pulmonary fibrosis [7,8,17]. Moreover, the absence of extracellular superoxide dismutase exacerbates conditions that lead to inflammation and pulmonary fibrosis [18]. Although these studies suggest that ROS contribute to lung damage and fibrosis, they do not clearly indicate the mechanisms of the antioxidant effect. That is, antioxidant compounds may attenuate oxidative damage caused directly by bleomycin [19], or they may limit the impact of ROS produced by phagocytes such as macrophages and neutrophils [5] and thus interfere with the inflammatory process. To clarify the role of ROS produced by phagocytes in the development of pulmonary fibrosis, we induced pulmonary fibrosis by i.n. bleomycin administration to p47^phox^-/- KO mice. Unlike antioxidant compounds, which nonspecifically target all ROS sources in the tissue, knocking out the p47^phox ^subunit of the NADPH oxidase complex shuts down only the main pathway of phagocytic ROS production. In this study, *in vitro *PMA stimulation of BAL cells from KO mice produced no detectable ROS, while BAL cells from WT mice did produce ROS, as previous studies of circulating neutrophils from these mice have shown [15].

Hydroxyproline content did not increase in the lungs of these KO mice. This finding reveals the absence of fibrosis and thus provides strong evidence that phagocytic ROS production is an important component of the fibrogenic environment.

Although no ROS production was detected in KO mice, their BAL gave evidence of an acute inflammatory response, as did that from WT mice: bleomycin administration elicited acute inflammation, characterized by an influx of neutrophils and associated with increased pro-MMP-9 activity. Moreover, the response of the KO mice to the bleomycin resembled the abnormal "exuberant" inflammation *in vivo *previously described in such KO mice [15,20,21]. It is, however, possible that this neutrophil influx and the large amounts of MMP-9 it releases have a protective effect against the development of pulmonary fibrosis. This exaggerated inflammatory response may be caused by defective down-regulation of the inflammatory process in these KO mice, perhaps due to the absence of ROS and the failure to degrade chemotactic signals [22].

This acute inflammation was accompanied by significantly elevated IL-6 levels in the BAL fluids of both strains of mice on the day after bleomycin administration, levels significantly higher in WT than KO mice. Given that IL-6 is secreted primarily by mononuclear cells, specifically macrophages, the difference between strains suggests that these cells are activated more weakly in the KO than in the WT mice. Moreover, in SP-D -/- alveolar macrophages, the NADPH oxidase inhibitor apocynin inactivates NF-kappa B, the transcription factor that regulates numerous proinflammatory responses, including IL-6 release [23]. Similarly, lipopolysaccharide-induced NF-kappa B activation is impaired in nuclear protein extracts of lung tissue from p47^phox^-/- KO mice [24]. This would explain why IL-6 response seems to be redox-sensitive in our experiment.

MMP induction, assessed by gelatinase release, has been reported in various cases of pulmonary fibrosis in human and experimental models [13,25,26]. We therefore analyzed the gelatinolytic activities of MMPs, in both BAL fluid and lung homogenate. MMP-9 and MMP-2 activities in the BAL fluid of the KO mice reveal an intense response to bleomycin. MMP-9 levels were highly correlated with neutrophil infiltration, while MMP-2 is known to be produced mainly by epithelial [27] and mesenchymal cells, such as fibroblasts, which are involved in collagen production and deposition [9,25]. This suggests that the exaggerated response observed in the BAL fluid of KO mice involves a wide spectrum of cell types. Surprisingly, at day 14, gelatinase profiles were different in the lung homogenates than in BAL fluids. Although MMP-2 activity was equivalent in BAL of both strains, MMP-2 activity increased substantially in the lung homogenate of the WT mice. One possible explanation is that the increased release of MMP-2 in the inner lung parenchyma may result from downstream events caused by phagocyte activation and ROS production during inflammation. This is consistent with another study, mentioned above, in which apocynin, an inhibitor of NADPH oxidase, also inhibited the release of MMP-9 and MMP-2 in SP-D -/- alveolar macrophages [23]. Moreover, Pardo et al. [28] report increased levels of gelatinases (MMP-2 and MMP-9) in isolated type II alveolar cells from hyperoxic rats; these increases are associated with alterations in the balance between MMPs and TIMPs and finally lead to diffuse alveolitis and its progression to pulmonary fibrosis.

It is thus difficult to reach a definitive conclusion at this time about the exact function of MMPs. They play a role in promoting tissue remodeling and counterbalancing excessive matrix deposition, but may also facilitate tissue damage and disruption. Their involvement in bleomycin-induced pulmonary fibrosis has been demonstrated with the inhibition of collagen deposition by bastimastat, a nonselective MMP-inhibitor [10]. Complete understanding of the dynamic process of remodeling nonetheless requires consideration of TIMPs, which are natural MMP inhibitors.

Increased TIMP-1 expression has been observed in lung extracts and in BAL fluids after bleomycin administration and after the transfer of the active TGF-beta gene to "fibrosis-prone" C57BL/6 mice [11,14]. In humans, increased levels of TIMP protein and RNA are observed in lungs of patients with idiopathic pulmonary fibrosis, and TIMP expression there exceeds that of MMP [12]. A reduced molar MMP/TIMP ratio seems to be a hallmark of pulmonary fibrosis, distinguishing it from other reversible interstitial lung diseases [26] and from chronic obstructive pulmonary disease (COPD) [29]. This ratio might be considered to be a "snapshot" of the dynamic matrix remodeling in lung tissue.

Interestingly, in our study, the pro-MMP-9/TIMP-1 ratio was significantly higher for KO than WT mice, at both day one and day 14. At day 1 this was due to the lower MMP-9 level and higher TIMP-1 level in the BAL from WT mice, and at day 14, only to the latter. The correlation of these levels with differences in hydroxyproline levels in the lungs of bleomycin-treated mice strongly suggests that a reduced molar pro-MMP-9/TIMP-1 ratio in BAL fluid is associated with collagen deposition, beginning as early as the inflammatory events at day 1 after bleomycin administration.

The usefulness of the pro-MMP-9/TIMP-1 ratio as a marker of fibrosis nonetheless requires discussion. Although a molar ratio appears to play a protective role against fibrotic changes, MMP-9 is considered primarily to be an inflammatory mediator released by leukocytes during acute inflammatory events to facilitate their progression across the basement membrane [30]. Moreover, MMP-9 depletion in KO mice does not substantially alter the extent of either pulmonary fibrosis or lung inflammation after bleomycin administration [31]. TIMPs may also counterbalance the activity of MMP-2 or other proteinases, such as collagenases. Ruiz et al. [32] recently observed that MMP-8 and MMP-13 RNA levels decreased and TIMP-1 RNA increased in the paraquat- and hyperoxia-induced pulmonary fibrosis rat model. Matrilysin (MMP-7), which can degrade various substrates, seems to have a crucial role in pulmonary fibrosis [33]. Finally, the exuberant neutrophil influx observed at day one in p47^phox^-/- KO mice could provide great amount of other kind of protease, such as serine proteases. Indeed, neutrophil elastase was shown to have an impact on the severity of bleomycin-induced pulmonary fibrosis [34,35]

## Conclusion

In summary, this study demonstrates that the inability of phagocytes from p47^phox^-/- KO mice to produce large quantities of ROS via the NADPH oxidase pathway inhibits the development of bleomycin-induced pulmonary fibrosis. This inhibition is associated with changes in IL-6 production and in the molar MMP-9/TIMP-1 ratio, both probably key factors in airway remodeling and fibrosis. These rapidity of these differences after bleomycin administration suggests that early inflammatory events and remodeling events may establish a favorable environment for further chronic fibrogenic processes.

## Abbreviations

**BAL: **Bronchoalveolar lavage

**IL: **interleukin

**KO: **knock out

**MMP: **matrix metalloproteinase

**TIMP: **tissue inhibitor of metalloproteinase

**ROS: **reactive oxygen species

**WT: **wild type

## Authors' contributions

BM, SN, OL, IG:and EB have made substantial contributions to acquisition and analysis of data

BM, SN, EB and VL have made substantial contributions to conception and design

BM, EB and VL have been involved in drafting the article

JMP and CPB have been involved in revising it critically for important intellectual content
